# Operating time for wire ligation with self‐ligating and conventional brackets: A standardized in vitro study

**DOI:** 10.1002/cre2.642

**Published:** 2022-08-26

**Authors:** Paolo M. Cattaneo, Michele Tepedino, Emilie B. Hansen, Anna R. Gram, Marie A. Cornelis

**Affiliations:** ^1^ Melbourne Dental School, Faculty of Medicine, Dentistry and Health Sciences University of Melbourne Carlton Australia; ^2^ Department of Biotechnological and Applied Clinical Sciences University of L'Aquila L'Aquila Italy; ^3^ Section of Orthodontics, Department of Dentistry and Oral Health Aarhus University Aarhus Denmark

**Keywords:** fixed appliance, operating time, self‐ligating brackets

## Abstract

**Objective:**

Operating time is one of the main advantages attributed from the literature to the use of self‐ligating brackets (SLB). The aim of this study is to investigate the time needed for a complete archwire change procedure with conventional brackets (CB) and SLBs in a standardized in vitro research setting, comparing operators with different expertise.

**Materials and Methods:**

Thirty‐three participants were divided into three equal groups: undergraduate students, postgraduate students, and orthodontists. Three sets of typodonts bonded with three types of brackets, including passive SLBs, active SLBs, and CBs using both steel and elastic ligatures were investigated. Operators had to insert, ligate, deligate, and remove wires in sets of typodonts representing an actual dentition before and after orthodontic treatment, mounted in phantoms. Archwire change procedure times were compared between the different bracket/ligation systems, between the before‐ and after‐treatment typodonts, and between operators.

**Results:**

There were significant differences between SLBs and CBs, the greatest difference being 11 min 16 s between passive SLBs and CBs ligated with metallic ligatures at T0, for the total archwire change procedure by the operators overall. For all the operators, there was a statistically significant difference in total archwire change procedure time between the systems. The undergraduate students were the slowest when using CBs, but they showed no significant difference compared to the other users when using SLBs.

**Conclusion:**

SLBs can offer a significant operating time reduction compared to CBs, and time saving is not dependent on the operator's experience and training.

## 1 INTRODUCTION

Self‐ligating brackets (SLBs) were introduced by Stolzenberg (1935) but only started to become popular in the late 90s because of a massive marketing strategy. Since then, many different designs and brands of SLBs became available. SLBs can be divided into two main categories: active SLBs, where the spring clip that closes the slot is designed to contact and exert pressure on the archwire, and passive SLBs, where the slot is closed by a sliding door that does not intrude into its lumen, providing any active contact or pressure onto the archwire. Manufacturers of both types of SLBs claimed that many advantages could derive from the use of their systems, including more physiological tooth movement due to the reduced friction between the slot and the archwire, reduced need for orthodontic extractions due to the promotion of new bone formation following the application of light and physiological forces, full and secure wire ligation, better sliding mechanics and less anchorage requirements, reduced treatment time with a lower number of appointments, improved ergonomics, reduced chairside time, improved patient comfort, and better oral hygiene (Berger & Byloff, 2001; Chen et al., 2010; Damon, 1998a, 1998b; Eberting et al., 2001; Harradine, 2003; Maijer & Smith, 1990). Years after the wide spreading of this technology–and its associated techniques–the scientific community started testing the companies' claims, which consequently were drastically revised. Current evidence suggests that faster alignment or space closure cannot be obtained with SLBs in a clinical setting, that alveolar bone expansion cannot be achieved (Cattaneo et al., 2011), and overall it is not supported by the scientific literature that SLBs achieve more efficient or better results than conventional brackets (CBs) (Chen et al., 2010; Yang et al., 2018).

On the other hand, the main reasons behind the introduction of SLBs were not related to the abovementioned claims, but they were originally proposed to reduce chairside time by using a built‐in closure mechanism that eliminates the need for an external ligature (Stolzenberg, 1935). There is evidence from a systematic review that this can be a real advantage of SLBs over CBs (Chen et al., 2010). In fact, a meta‐analysis of data pooled from two studies (Harradine, 2001; Turnbull & Birnie, 2007) reported that using passive SLBs (Damon, Ormco, Glendora, Calif, USA) allows saving 20 s per arch during slot opening compared to deligating CBs with elastomeric ligatures, while there was no statistically significant difference during slot closure/ligation (Chen et al., 2010). However, these results are based on only two studies at moderate risk of bias, with one of them (Harradine, 2001) reporting chairside time only as a secondary outcome. Out of the 16 studies included in the qualitative synthesis by Chen et al. (2010), only a few studies (Maijer & Smith, 1990; Paduano et al., 2008; Turnbull & Birnie, 2007) had the chairside time for an archwire change as the primary outcome, while in the other studies, time was only assessed as a secondary outcome, sometimes with a limited description of the methodology used (Shivapuja & Berger, 1994; Voudouris, 1997). Interestingly, the article from Shivapuja and Berger (1994) was removed from the meta‐analysis regarding the ligation part, as it was wrongly considered an “in vitro” study: as a matter of fact, based on the results from the clinical observation of the ligation time, it was reported that “dramatically less chairtime for arch wire removal and insertion” could be seen in a clinical setting (i.e., more than 1.5 and 7 min per arch in comparison to remove and replace elastomeric and steel ligatures respectively). The existing literature on chairside time for wire ligation is based on studies conducted in a clinical environment on actual patients (Berger & Byloff, 2001; Harradine, 2001; Maijer & Smith, 1990; Paduano et al., 2008; Shivapuja & Berger, 1994; Turnbull & Birnie, 2007), thus with the differences in malocclusion possibly altering the time for archwire change, and it is relatively old, thus based on outdated SLB bracket types. Moreover, according to Turnbull and Birnie (2007) shorter times were measured for larger wires, used at later stages of treatment when teeth are well aligned. Similarly, other studies were done on aligned dentitions, which, again, do not reflect the daily clinical practice for the same reason (Berger & Byloff, 2001; Paduano et al., 2008). Additionally, differences in time for archwire change (i.e., insert wire, ligate, deligate, and remove wire) between operators have never been assessed.

To fill the gap of the limitations of previous studies, and to acknowledge the technological improvements in SLB design, the aim of the present study is to assess the time needed for changing archwires using passive SLB, active SLB, CB with elastomeric ligatures, and CB with metallic ligatures, in a standardized research setting on crowded and on well‐aligned arches, by operators with different levels of experience. The null hypothesis was that there is no difference between bracket systems, between operators, and between crowded and well‐aligned arches.

## 2 MATERIALS AND METHODS

### 2.1 Experimental setup

**Figure 1:**
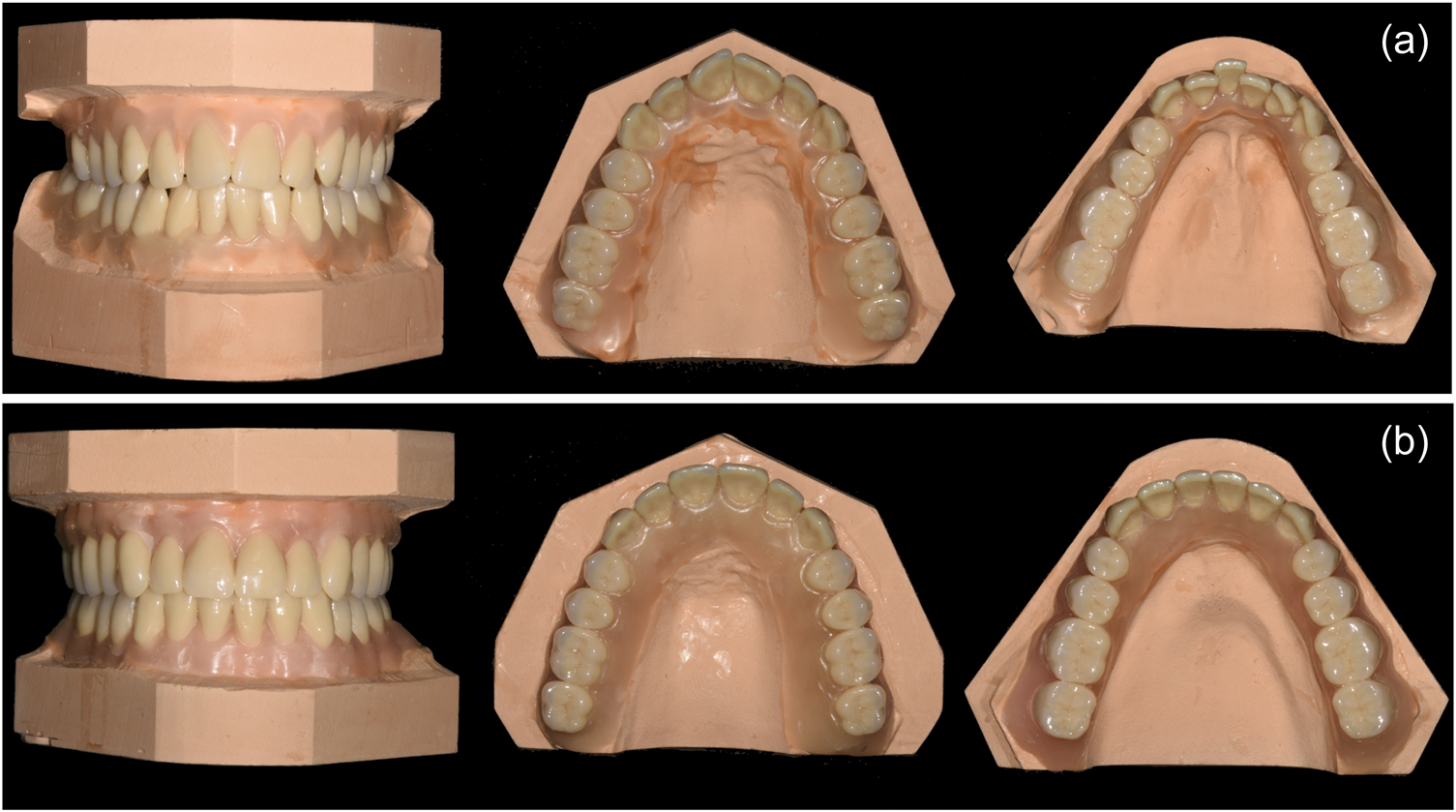
Models of the initial malocclusion (a, before treatment) and of the aligned arches (b, after treatment) obtained from the dental casts of a real patient.

The fully anonymized pre‐ and posttreatment digital models (T0 and T1 models, respectively) of an actual patient with Class I and moderate crowding, treated with SLB, were printed, and three identical sets of pre‐ (T0) and posttreatment (T1) typodonts reproducing the initial malocclusion and final occlusion were manufactured for the purpose of the research project (Figure 1 and Supporting Information: Figure 1). Three different brackets systems were bonded on each set of T0 and T1 typodonts from the second right molar to second left molar: passive SLBs (Damon Q ‐ 0.022″ slot ‐ Ormco, SDS, Orange, CA); Active SLBs (Empower – according to manufacturer's description: 0.018″ slots on incisors and canines, 0.022″ slots on premolars and molars – American Orthodontics, Sheboygan, WI); CBs (Master Series, Low Profile ‐ 0.022″ slot – American Orthodontics, Sheboygan, WI). At T0, upper and lower 0.014″ NiTi wires were selected for the three typodonts, while at T1 upper and lower 0.017 × 0.025″ stainless steel wires were used for the three typodonts. The typodonts were mounted in patient simulators in order to replicate clinical conditions (Supporting Information: Figure 2). Regarding the SLBs, the specific instruments developed by the manufacturers (Ormco for Damon Q brackets and American Orthodontics for Empower brackets) were used for opening and closing the slots, while conventional pliers and instruments were used to ligate the CBs with elastomeric or metallic ligatures.

### 2.2 Procedures

Three groups of operators were recruited for the study: undergraduate dental students (in their third semester of orthodontics curriculum teaching), postgraduate students in orthodontics (second and third year), and certified orthodontists. A convenience sample of 11 operators for each group was recruited. All operators were students or employees at the Section of Orthodontics, Department of Dentistry and Oral Health, Aarhus University, Denmark, who volunteered to participate. The certified orthodontists had 8.5 years (±4.8) of experience. The postgraduate students were acquainted with the use of CBs and SLBs, as those brackets were routinely used during their clinical training. The undergraduate students had previously performed bonding and ligation procedures on patients as part of their education, and performed a training session on the typodonts, before the experimental session.

Each operator was asked, for the three sets of T0 and T1 typodonts, in a random order, to perform the following four archwire change steps: 1) insert the upper and lower wires; 2) ligate the upper and lower wires (for CBs) or close the upper and lower brackets' slots (for SLBs); 3) remove all ligatures (for CBs) or open all brackets' slots (for SLBs); and 4) remove the upper and lower wires. For the T0 and T1 typodonts with CBs, this procedure was performed twice: once with elastomeric ligatures (Sani‐Tie; Dentsply GAC International Inc., Bohemia, USA) and once with metallic ligatures (Thru Chrome; Rocky Mountain Orthodontics, Colorado, USA).

The time to perform Steps 1, 2, 3, and 4 was recorded separately with a digital stopwatch to the closest second. The total time for all four steps was also logged.

### 2.3 Statistical analysis

Statistical analysis was performed with SPSS version 21.0 for Windows (IBM SPSS Statistics for Windows, Version 21.0, Armonk, NY).

Descriptive statistics were computed for the time used by all operators, and by all operator groups, on the T0 and T1 typodonts with different bracket systems. A Shapiro–Wilk normality test was used to evaluate the data distribution. To compare the time needed to perform Steps 1–4 on the T0 and T1 typodonts, independent sample *T*‐tests or Mann–Whitney *U* tests were used, depending on data distribution. To compare the total time (for all four steps) between bracket systems (for all operators, and by operator group) and between operators, one‐way analysis of variance (ANOVA) was used. To test the assumption of data homoscedasticity, Levene's tests were used, and if unequal variances were found, a Welch ANOVA was performed. Post hoc pairwise comparisons were performed with a Tukey honestly significant difference or a Games–Howell test, depending on the homogeneity of variances. A first‐type error was set at *p* = .05 for all the tests.

## 3 RESULTS

The null hypothesis was rejected since time differences were observed between different bracket systems, between different operator groups, and between crowded (T0) and well‐aligned (T1) arches. Descriptive statistics are reported in Supporting Information: Tables 1 and 2. When comparing the T0 and T1 typodonts, archwire removal was statistically significant longer at T1 for all bracket systems (Table 3). Slot closure with active and passive SLBs was also faster at T1 compared to T0 (Table 1).

**Table 1:** Comparison of time for both arches by all operators (*n* = 33), between T0 and T1 typodonts, for the different bracket systems

System	Operation	T0 median	T1 median	*U*‐statistics	*p*‐Value
Active SLB	Archwire insertion	55.0	68.0	684.5	.073
	Ligation/closure	67.0	50.0	336.0**	.007
	Deligation/opening	47.0	49.0	533.0	.883
	Archwire removal	6.0	16.0	945.5**	<.001
	Total time	188.0	200.0	608.5	.412
Passive SLB	Archwire insertion	66.0	60.0	483.0	.430
	Ligation/closure	51.0	42.0	360.5*	.018
	Deligation/opening	34.0	34.0	594.5	.520
	Archwire removal	6.0	8.0	747.0**	.009
	Total time	166.0	151.0	497.5	.547
CB elastomeric ligatures	Archwire insertion	60.0	49.0	346.5*	.011
	Ligation/closure	282.0	252.0	447.0	.211
	Deligation/opening	74.0	74.0	522.0	.773
	Archwire removal	5.0	8.0	725.5*	.020
	Total time	447.0	388.0	440.0	.180
CB metallic ligatures	Archwire insertion	50.0	46.0	465.0	.307
	Ligation/closure	476.0	472.0	487.0	.461
	Deligation/opening	182.0	172.0	459.5	.276
	Archwire removal	4.0	10.0	750.0**	.008
	Total time	762.0	676.0	458.0	.267

*Note*: Mann–Whitney *U* test.

Abbreviations: CB, conventional bracket; SLB, self‐ligating bracket.

* Statistically significant with *p* < .05.

** Statistically significant with *p* < .01.

When pooling together all operators, a statistically significantly shorter time was found for SLBs compared to CBs, with regard to ligating/slot closure and total time, both with the T0 and T1 typodonts (Table 2). Ligating/slot closure at T0, deligating/slot opening (at T0 and at T1), archwire removal at T1, and total time at T1 were also shorter for passive SLBs compared to active SLBs. The step that provided the greatest time saving was the closure of the slots/ligating with SLBs compared to CBs, while a small difference of about 17–19 s was seen between active and passive SLBs.

**Table 2:** Comparison of time spent by all operators (*n* = 33), between the different bracket systems

Treatment stage	Operation	Levene statistic (*p*‐value)	*F* a	*p*‐Value	Post hoc comparison^b^					
					Active SLB versus passive SLB	Active SLB versus CB elastomeric ligatures	Active SLB versus CB metallic ligatures	Passive SLB versus CB elastomeric ligatures	Passive SLB versus CB metallic ligatures	CB elastomeric ligatures versus CB metallic ligatures
T0	Archwire insertion	.434	1.25	.295	10.2 (0.563)	0.5 (1.000)	4.2 (0.949)	10.8 (0.519)	14.5 (0.258)	3.7 (0.965)
	Ligation/closure	<.001	85.87**	<.001	19.0** (0.001)	−252.7** (<0.001)	−464.8** (<0.001)	−271.6** (<0.001)	−483.8** (<0.001)	−212.1** (<0.001)
	Deligation/opening	<.001	67.65**	<.001	20.6** (<0.001)	−32.6** (<0.001)	−152.3** (<0.001)	−53.2** (<0.001)	−172.9** (<0.001)	−119.7** (<0.001)
	Archwire removal	.003	0.62	.602	0.5 (0.965)	0.6 (0.948)	−3.1 (0.694)	0.1 (1.000)	3.6 (0.588)	−3.7 (0.570)
	Total time	.021	75.88**	<.001	29.8 (0.148)	−284.1** (<0.001)	−615.9** (<0.001)	−314.0** (<0.001)	−645.8** (<0.001)	−331.8** (<0.001)
T1	Archwire insertion	.006	6.77**	<.001	12.5 (0.432)	30.1** (0.002)	25.4* (0.036)	17.6* (0.014)	12.5 (0.330)	−4.7 (0.902)
	Ligation/closure	<.001	74.91**	<.001	16.7 (0.119)	−223.8** (<0.001)	−441.1** (<0.001)	−240.5** (<0.001)	−457.8** (<0.001)	−217.2** (<0.001)
	Deligation/opening	<.001	40.27**	<.001	21.1* (0.020)	−23.8 (0.051)	−139.0** (<0.001)	−44.9** (<0.001)	−160.1** (<0.001)	−115.2** (<0.001)
	Archwire removal	.355	4.38**	.006	7.27* (0.023)	8.39** (0.006)	5.45 (0.138)	1.1 (0.970)	−1.8 (0.888)	−2.9 (0.648)
	Total time	<.001	58.34**	<.001	57.6** (0.003)	−209.1** (<0.001)	−549.2** (<0.001)	−266.7** (<0.001)	−606.8** (<0.001)	−340.1** (<0.001)

*Note*: One‐way ANOVA and post hoc tests.

Abbreviations: ANOVA, analysis of variance; CB, conventional bracket; HSD, honestly significant difference; SLB, self‐ligating bracket.

^a^ If the assumption of homogeneity of variances was not respected, the data shown are from Welch ANOVA.

^b^ Mean difference expressed in seconds (*p*‐value) from the Tukey HSD test or Games–Howell test depending on the homogeneity of variances.

* Statistically significant with *p* < .05.

** Statistically significant with *p* < .01.

**Figure 2:**
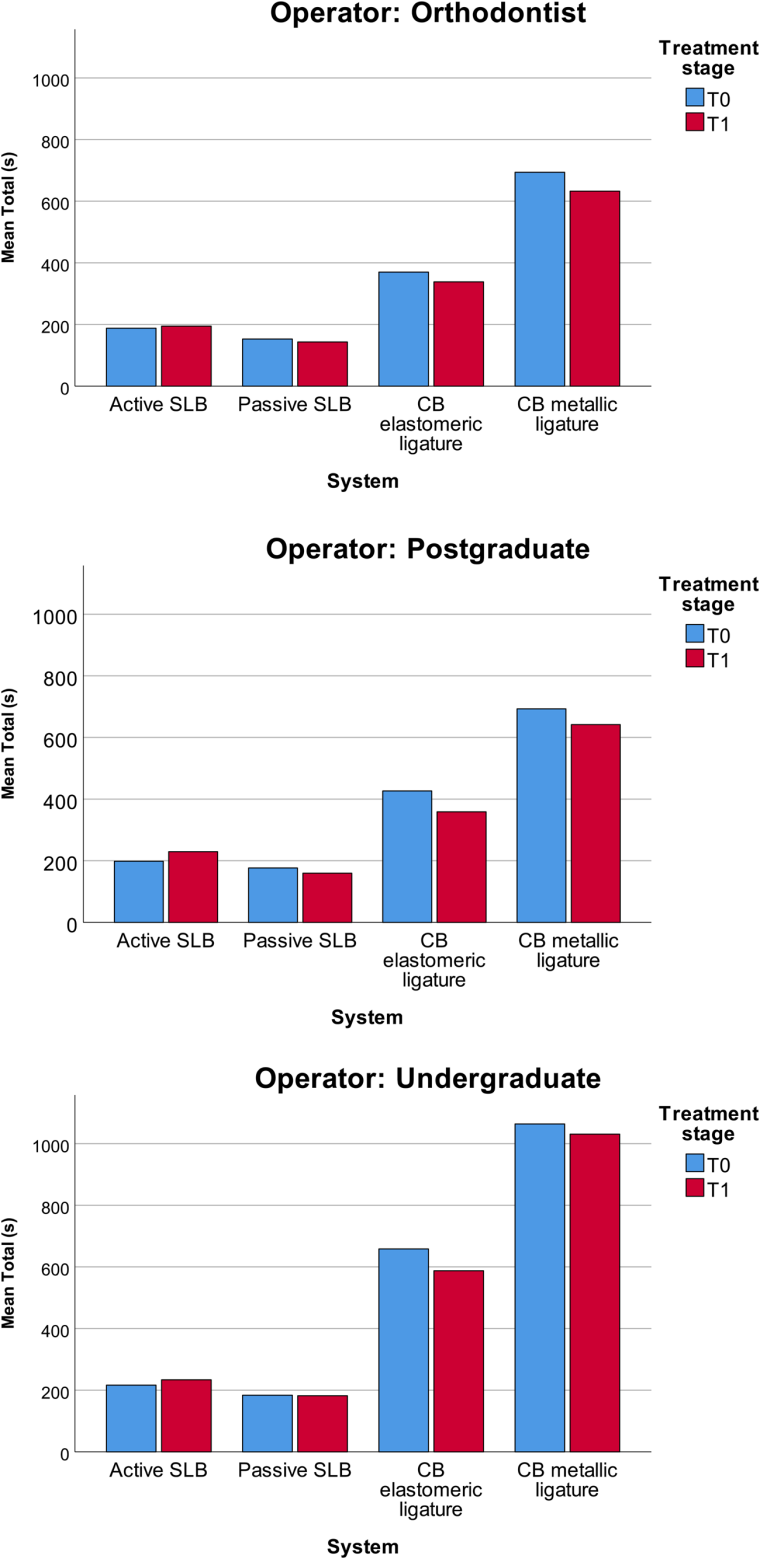
Histograms with the mean total time employed by each operator group for the different bracket systems. CB, conventional bracket;  SLB, self‐ligating bracket.

The data divided by operator groups are reported in Table 3 and in Figure 2.

**Table 3:** Comparison of time spent by the different operator groups (*n* = 11 per group), between the different bracket systems

Treatment stage	Operator group	Operation	Levene statistic (*p*‐value)	*F* a	*p‐*Value	Post hoc comparison^b^					
						Active SLB versus passive SLB	Active SLB versus CB elastomeric ligature*s*	Active SLB versus CB metallic ligatures	Passive SLB versus CB elastomeric ligatures	Passive SLB versus CB metallic ligatures	CB elastomeric ligatures versus CB metallic ligatures
T0	Orthodontists	Archwire insertion	.168	0.031	.992	−3.4 (0.991)	−1.0 (1.000)	−1.0 (1.000)	2.4 (0.997)	2.4 (0.997)	0.0 (1.000)
		Ligation/closure	<.001	76.215**	<.001	22.4* (0.038)	−168.2** (<0.001)	−376.2** (<0.001)	−190.6** (<0.001)	−398.6** (<0.001)	−208.0** (<0.001)
		Deligation/opening	<.001	45.559**	<.001	13.8 (0.085)	−15.0 (0.055)	−129.8** (<0.001)	−28.8** (<0.001)	−143.6** (<0.001)	−114.8** (<0.001)
		Archwire removal	.105	0.536	.660	2.1 (0.690)	2.0 (0.719)	1.0 (0.952)	−0.1 (1.000)	−1.1 (0.939)	−1.0 (0.952)
		Total time	.002	62.770**	<.001	35.0 (0.197)	−182.2** (<0.001)	−506.0** (<0.001)	−217.2** (<0.001)	−541.0** (<0.001)	−323.8** (<0.001)
	Postgraduate students	Archwire insertion	.206	1.569	.212	−15.3 (0.511)	1.7 (0.999)	7.4 (0.904)	17.0 (0.417)	22.7 (0.179)	5.7 (0.953)
		Ligation/closure	<.001	36.692**	<.001	13.8 (0.130)	−207.6** (0.001)	−352.6** (<0.001)	−221.4** (<0.001)	−366.4** (<0.001)	−145.0 (0.065)
		Deligation/opening	<.001	47.925**	<.001	23.8** (0.002)	−20.9 (0.118)	−137.7** (<0.001)	−44.7** (0.001)	−161.5** (<0.001)	−116.8** (<0.001)
		Archwire removal	.001	0.758	.530	−0.6 (0.993)	−1.4 (0.938)	−11.4 (0.468)	−0.7 (0.992)	−10.7 (0.520)	−10.0 (0.573)
		Total time	<.001	35.678**	<.001	21.7 (0.609)	−228.2** (0.002)	−494.3** (<0.001)	−249.9** (0.001)	−516.0** (<0.001)	−266.1** (0.005)
	Undergraduate students	Archwire insertion	.581	0.388	.762	−12.1 (0.901)	0.9 (1.000)	6.3 (0.984)	13.0 (0.881)	18.4 (0.725)	5.4 (0.990)
		Ligation/closure	<.001	61.265**	<.001	20.6 (0.268)	−382.2** (<0.001)	−665.6** (<0.001)	−402.8** (<0.001)	−686.3** (<0.001)	−283.4* (0.041)
		Deligation/opening	<.001	21.098**	<.001	24.3* (0.042)	−61.9* (0.012)	−189.3** (0.004)	−86.2** (0.001)	−213.5** (0.002)	−127.4 (0.051)
		Archwire removal	.117	0.522	.670	0.0 (1.000)	1.2 (0.795)	1.1 (0.831)	1.2 (0.795)	1.1 (0.831)	−0.1 (1.000)
		Total time	<.001	43.746**	<.001	32.8 (0.772)	−442.0** (<0.001)	‐847.5** (<0.001)	−474.8** (<0.001)	‐880.4** (<0.001)	−405.5* (0.021)
T1	Orthodontists	Archwire insertion	.019	1.364	.281	21.5 (0.580)	30.3 (0.306)	29.3 (0.372)	8.7 (0.631)	7.7 (0.838)	−1.0 (1.000)
		Ligation/closure	<.001	73.694**	<.001	8.4 (0.531)	−162.5** (<0.001)	−343.6** (<0.001)	−170.9** (<0.001)	−352.0** (<0.001)	−181.1** (<0.001)
		Deligation/opening	.001	34.707**	<.001	15.6* (0.028)	−17.8* (0.039)	−126.9** (<0.001)	−33.4** (<0.001)	−142.5** (<0.001)	−109.1** (<0.001)
		Archwire removal	.384	1.389	.260	5.7 (0.340)	6.1 (0.287)	3.2 (0.783)	0.4 (1.000)	−2.5 (0.875)	−2.9 (0.825)
		Total time	.002	47.611**	<.001	51.3 (0.177)	−144.0** (0.001)	−438.1** (<0.001)	−195.3** (<0.001)	−489.4** (<0.001)	−294.1** (<0.001)
	Postgraduate students	Archwire insertion	.197	3.787*	.018	24.7 (0.296)	44.5* (0.013)	34.4 (0.079)	19.8 (0.489)	9.6 (0.898)	−10.2 (0.883)
		Ligation/closure	<.001	91.756**	<.001	17.8 (0.126)	−164.0** (<0.001)	−324.6** (<0.001)	−181.8** (<0.001)	−360.4** (<0.001)	−178.6** (<0.001)
		Deligation/opening	<.001	30.599**	<.001	17.4* (0.011)	−20.5* (0.031)	−115.6** (<0.001)	−38.0** (<0.001)	−133.1** (<0.001)	−95.1** (0.001)
		Archwire removal	.382	2.046	.123	9.8 (0.258)	10.4 (0.216)	11.4 (0.147)	0.5 (1.000)	1.6 (0.989)	1.1 (0.997)
		Total time	<.001	39.703**	<.001	69.8** (0.004)	−129.6** (0.002)	−412.4** (<0.001)	−199.4** (<0.001)	−482.3** (<0.001)	−282.8** (0.001)
	Undergraduate students	Archwire insertion	.169	1.631	.197	−8.7 (0.896)	15.4 (0.604)	12.4 (0.749)	24.2 (0.226)	21.2 (0.335)	−3.0 (0.995)
		Ligation/closure	<.001	40.523**	<.001	23.9 (0.569)	−344.9** (<0.001)	−636.9** (<0.001)	−368.8** (<0.001)	−660.8** (<0.001)	−292.0* (0.024)
		Deligation/opening	.001	9.809**	.001	30.2 (0.458)	−33.1 (0.575)	−174.4* (0.043)	−63.3* (0.013)	−204.6* (0.016)	−141.4 (0.111)
		Archwire removal	.095	1.695	.183	6.3 (0.485)	8.7 (0.206)	1.7 (0.979)	2.4 (0.943)	−4.5 (0.727)	−7.0 (0.389)
		Total time	<.001	23.938**	<.001	51.6 (0.502)	−353.8** (0.002)	−797.2** (<0.001)	−405.4** (0.001)	−848.8** (<0.001)	−443.4* (0.034)

*Note*: One‐way ANOVA and post hoc tests.

Abbreviations: ANOVA, analysis of variance; CB, conventional bracket; HSD, honestly significant difference; SLB, self‐ligating bracket.

^a^ If the assumption of homogeneity of variances was not respected, the data shown are from Welch ANOVA.

^b^ Mean difference expressed in seconds (*p*‐value) from the Tukey HSD test or Games–Howell test depending on the homogeneity of variances.

* Statistically significant with *p* < .05.

** Statistically significant with *p* < .01.

The undergraduate students were significantly slower compared to both orthodontists and postgraduate students when using CBs with either elastomeric or metallic ligatures, but not when using active or passive SLBs (Table 4).

**Table 4:** Comparison of time between the different operator groups

Treatment stage	System	Operation	Levene statistic (*p*‐value)	*F* a	*p*‐Value	Post hoc comparison^b^		
						Orthodontists versus postgraduate students	Orthodontists versus undergraduate student*s*	Post‐ versus undergraduate students
T0	Active SLB	Archwire insertion	.071	0.568	.573	−4.6 (0.952)	−16.0 (0.561)	−11.4 (0.745)
		Ligation/closure	.065	0.875	.427	3.9 (0.916)	−8.7 (0.649)	−12.6 (0.411)
		Deligation/opening	.149	0.735	.488	‐9.9 (0.459)	−5.8 (0.760)	4.1 (0.873)
		Archwire removal	.032	1.225	.320	0.0 (1.000)	1.9 (0.469)	1.9 (0.490)
		Total time	.031	0.419	.664	−10.6 (0.808)	−28.6 (0.666)	−18.0 (0.833)
	Passive SLB	Archwire insertion	.333	1.718	.197	−16.5 (0.452)	−24.7 (0.181)	−8.2 (0.820)
		Ligation/closure	.123	1.240	.304	−4.7 (0.763)	−10.5 (0.273)	−5.8 (0.665)
		Deligation/opening	.262	1.681	.203	0.1 (0.999)	4.6 (0.260)	4.5 (0.273)
		Archwire removal	.091	1.508	.238	−2.7 (0.281)	−0.2 (0.994)	2.5 (0.329)
		Total time	.244	1.351	.274	−23.9 (0.454)	−30.8 (0.276)	−6.9 (0.934)
	CB elastomeric ligatures	Archwire insertion	.841	1.038	.366	−1.9 (0.982)	−14.1 (0.391)	−12.2 (0.493)
		Ligation/closure	.315	15.197**	<.001	−35.5 (0.694)	−222.7** (<0.001)	−187.2** (<0.001)
		Deligation/opening	.001	7.037**	.007	−15.8 (0.152)	−52.7* (0.016)	−36.9 (0.109)
		Archwire removal	.090	2.826	.075	−3.4 (0.214)	1.1 (0.843)	4.4 (0.074)
		Total time	.145	13.028**	<.001	−56.6 (0.616)	−288.4** (<0.001)	−231.8** (0.002)
	CB metallic ligatures	Archwire insertion	.554	0.391	.680	3.8 (0.963)	−8.7 (0.821)	−12.5 (0.668)
		Ligation/closure	.019	5.779*	.011	27.4 (0.856)	−298.2* (0.017)	−325.6* (0.011)
		Deligation/opening	.020	1.293	.298	−17.8 (0.665)	−65.3 (0.312)	−47.4 (0.537)
		Archwire removal	.002	2.024	.161	−12.4 (0.273)	2.0 (0.609)	14.4 (0.183)
		Total time	.005	5.062*	.018	1.1 (1.000)	−370.2* (0.020)	−371.3* (0.021)
T1	Active SLB	Archwire insertion	.475	0.835	.444	−13.1 (0.722)	8.6 (0.867)	21.7 (0.415)
		Ligation/closure	<.001	2.760	.091	−10.6 (0.393)	−35.8 (0.119)	−25.2 (0.338)
		Deligation/opening	.122	0.334	.718	−5.8 (0.937)	−13.7 (0.697)	−7.9 (0.886)
		Archwire removal	.261	0.911	.413	−5.2 (0.593)	1.6 (0.948)	6.8 (0.410)
		Total time	.101	0.803	.458	−34.7 (0.568)	−39.3 (0.487)	−4.5 (0.990)
	Passive SLB	Archwire insertion	.031	1.963	.168	−9.9 (0.483)	−21.6 (0.171)	−11.7 (0.603)
		Ligation/closure	.040	2.153	.144	−1.2 (0.976)	−20.3 (0.133)	−19.1 (0.158)
		Deligation/opening	.478	1.592	.220	−4.0 (0.362)	0.8 (0.957)	4.8 (0.234)
		Archwire removal	.375	0.289	.751	−1.1 (0.966)	2.2 (0.873)	3.3 (0.738)
		Total time	.381	2.598	.091	−16.2 (0.618)	−38.9 (0.076)	−22.7 (0.393)
	CB elastomeric ligatures	Archwire insertion	.663	0.447	.644	1.2 (0.989)	−6.2 (0.742)	−7.4 (0.656)
		Ligation/closure	.032	9.593**	.001	−12.1 (0.857)	−218.2** (0.002)	−206.1** (0.003)
		Deligation/opening	.045	1.937	.173	−8.5 (0.434)	−29.0 (0.233)	−20.4 (0.473)
		Archwire removal	.790	1.632	.212	−0.9 (0.953)	4.3 (0.356)	5.2 (0.225)
		Total time	.075	10.052**	<.001	−20.4 (0.942)	−249.1** (0.001)	−228.7** (0.002)
	CB metallic ligatures	Archwire insertion	.424	0.190	.828	−8.0 (0.858)	−8.2 (0.852)	−0.2 (1.000)
		Ligation/closure	.003	7.954**	.003	−9.6 (0.968)	−329.1** (0.004)	−319.4** (0.005)
		Deligation/opening	.013	0.666	.526	5.4 (0.973)	−61.3 (0.545)	−66.7 (0.489)
		Archwire removal	.087	0.285	.754	3.1 (0.781)	0.2 (0.999)	−2.9 (0.803)
		Total time	.003	4.345*	.028	−9.1 (0.991)	−398.4* (0.029)	−389.3* (0.034)

*Note*: One‐way‐way ANOVA and post hoc test.

Abbreviations: ANOVA, analysis of variance; CB, conventional bracket; HSD, honestly significant difference; SLB, self‐ligating bracket.

^a^ If the assumption of homogeneity of variances was not respected, the data shown are from Welch ANOVA.

^b^ Mean difference expressed in seconds (*p*‐value) from Tukey HSD test or Games–Howell test depending on the homogeneity of variances.

* Statistically significant with *p* < .05.

** Statistically significant with *p* < .01.

## 4 DISCUSSION

The influence of crowding on time for archwire change procedures has not been previously reported in the literature. This represents a major element of novelty of the present work, together with the effect of the operator's expertise on archwire change procedures time. The total archwire change time was not different at the beginning (T0) and at the end of treatment (T1) for the four tested bracket systems. However, T0 and T1 times were different for some specific tasks: removing the larger 0.017 × 0.025″ stainless steel wire at T1 required more time than the 0.014″ NiTi wire at T0, with every bracket system. With both SLB systems, the time needed for slot closure was shorter at T1, probably because at T0 some extra time was needed to seat the archwire in the slot before closing the clip. It is possible to partially compare the present results with those of Turnbull & Birnie (Turnbull & Birnie, 2007) – although with some limitations because the latter authors measured the ligation time on actual patients with different types of malocclusion – who found that ligating small round archwires required more time than large rectangular stainless steel archwires with passive SLBs.

Looking at the overall archwire change procedure time of the four bracket systems, without any distinction between operators and arch alignment stage, passive SLBs showed a shorter deligation time compared to active SLBs (Table 2). Both SLB systems granted a significant saving of time compared to CBs: about 4–5 min per patient with elastomeric ligatures, and about 10 min per patient with metallic ligatures, which was the slowest setting, as expected. Inserting an archwire on aligned arches (T1) into active or passive SLBs requires more time than into CBs, but the greatest difference calculated was only 30 s. Previous studies agreed that SLBs offer chairside time saving compared to CBs, even if those studies are not directly comparable due to heterogeneity in methods used and the differences related to the examination of real patients or typodonts. Turnbull and  Birnie (2007), for example, recorded a mean ligation time per arch of 46.3 s for passive SLBs and 98.4 s for CBs with elastomeric ligatures. Paduano et al. (2008) reported in a clinical study a mean time per arch of 22 s to close the clips of active and passive SLBs, 124 s to ligate CBs with elastomeric ligatures, and 183 s to ligate CBs with metallic ligatures, suggesting a time saving of 2–3 min (Paduano et al., 2008). According to Berger and Byloff (2001), closing the slots of different types of SLBs in both arches required from 18 to 55 s on actual patients, while ligating CBs required 2 min 32 s–2 min 40 s with elastomeric ligatures, and 9 min 32 s–11 min 23 s with metallic ligatures. In contrast to those results as well as the results of the present study, a systematic review with meta‐analysis of studies performed on patients reported a time saving of only 20 s per arch when opening the slides of passive SLBs compared to CBs, while the difference of 30 s during slot closure/ligation was not statistically significant (Chen et al., 2010). However, it must be underlined that the results of this meta‐analysis are based upon two studies only, one reporting chairside time as a secondary outcome and presenting a limited explanation of setting and methods (Harradine, 2001), thus leaving only one study focused on ligation time (Turnbull & Birnie, 2007). These data seem unrealistic in light of the results of the present study, where a standardized setting allowed to isolate multiple variables like arch crowding, operator experience, and the single procedures carried out during an archwire change procedure. From the results of the present study, it can be concluded that an average operator using CBs with elastomeric ligatures can save slightly more than 4 min with active SLBs, during a normal appointment where the archwires are removed for reactivation or changed. An additional 43 s per patient can be saved if using passive SLBs. This difference gets even larger if metallic ligatures are considered: using SLBs will save about 10 min per patient. Such a time difference can be really meaningful in clinical practice, considering that a typical orthodontic visit lasts from 15 to 20 min. The time saved can be used to talk with the patient to improve his/her cooperation and motivation, or to increase the number of patients seen during working hours: in the first case, the patient's perceived quality of treatment might be improved, while in the latter, the higher cost of SLBs can be justified by improving the revenue per hour of the orthodontic practice.

When looking at the archwire change procedure time of different bracket systems stratified by operator experience, an experienced operator was able to save 1 min 43 s per arch with passive SLBs against CBs with elastomeric ligatures, and 4 min 18 s per arch with passive SLBs against CBs with metallic ligatures, an unexperienced operator was able to save even more time (about 3 min 40 s per arch with passive SLBs against CBs with elastomeric ligatures, and about 7 min 12 s per arch with passive SLBs against CBs with metallic ligatures).

Comparing the time spent by different operators, no significant differences were found between orthodontists and postgraduate students. On the other hand, the undergraduate students were significantly slower than orthodontists and postgraduates using CBs, using about 2 min more with elastomeric ligatures and about 3 min more with metallic ligatures. Interestingly, there was no difference in total archwire change time with SLBs between the three operator groups, suggesting that SLBs do not require particular training and even less experienced operators can use them efficiently. This is a very interesting aspect, which never emerged before, since the previous studies considered only experienced and well‐trained operators (Berger & Byloff, 2001; Harradine, 2001; Maijer & Smith, 1990; Paduano et al., 2008; Turnbull & Birnie, 2007). This means, for example, an untrained dental assistant can change an archwire being as time‐efficient as an experienced one.

While the emerging evidence from randomized clinical trials and systematic reviews suggests no significant advantage of SLBs over CBs in expanding the transversal dimension, space closure, treatment time and efficiency (Yang et al., 2018), a number of total appointments, patient discomfort during initial alignment, (Čelar et al., 2013), or improved oral hygiene (do Nascimento et al., 2014), the true convenience offered by SLBs is a significantly reduced operating time.

Regarding the limitations of the present study, the use of typodonts and mannequins instead of real patients, while giving the opportunity to standardize the procedures and limit the confounders, produced slightly different results from a real clinical situation; for example, while cutting the metallic ligatures, the operators did not need to take care of the soft tissues to avoid any injuries, or to lose ligatures in the oral cavity. In addition, in the present setting, the operators were working alone, and the presence of a dental assistant preparing the metallic or elastomeric ligatures could have reduced the time measured for CBs. On the other hand, this setting allowed us to concentrate on the archwire change procedure, by removing all external factors, thus making the main findings of the present research valid. Finally, the use of a 0.017 × 0.025″ archwire at T1 might be considered undersized for passive SLBs, (Savoldi et al., 2017) but was the closest to full size for the bidimensional active SLBs: choosing two different wires would add a confounding factor, yet, the insertion time was not significantly different between active and passive SLBs.

## 5 CONCLUSION

A statistically and clinically significant shorter archwire change time is needed for SLBs compared to CBs (with either metallic or elastomeric ligatures). The greatest time saving was of over 5 min per arch and was achieved by using passive SLBs compared to CBs with metallic ligatures. Archwire change time was statistically significantly shorter for passive SLBs compared to active SLBs, but only on well‐aligned arches. Less experienced operators are slower with CBs compared to more experienced operators. By contrast, when using SLBs, there was no difference in total archwire change procedure time between operators with different levels of experience.

## AUTHOR CONTRIBUTIONS

Paolo M. Cattaneo conceived the project, designed the study protocol, and revised the manuscript draft. Michele Tepedino wrote the manuscript draft and performed the statistical analysis. Emilie B. Hansen and Anna R. Gram collected the data and drafted the work. Marie A. Cornelis supervised the project and revised the manuscript draft. Paolo M. Cattaneo and Michele Tepedino contributed equally to the present work and are both listed as the first authors. All authors read and approved the final version of the present manuscript.

## CONFLICT OF INTEREST

The authors declare no conflict of interest.

## Supporting information

Supplementary information.

## Data Availability

The datasets used and/or analyzed during the current study are available from the corresponding author on reasonable request.
