# Supplementary material for: Operating time for wire ligation with self‐ligating and conventional brackets: A standardized in vitro study
Source: Clin Exp Dent Res. 2022 Aug 26;8(6):1456–66. doi: 10.1002/cre2.642 (PMC9760137; doi:10.1002/cre2.642)
Supplement: Supplementary file 1 — Supplementary information. [file CRE2-8-1456-s001.docx]

Supporting figure 1

**SUPPORTING INFORMATION**

**Supporting figure 1.** Typodonts manufactured from the before and after treatment models. **A**, passive SLBs. **B**, active SLBs. **C**, conventional brackets.


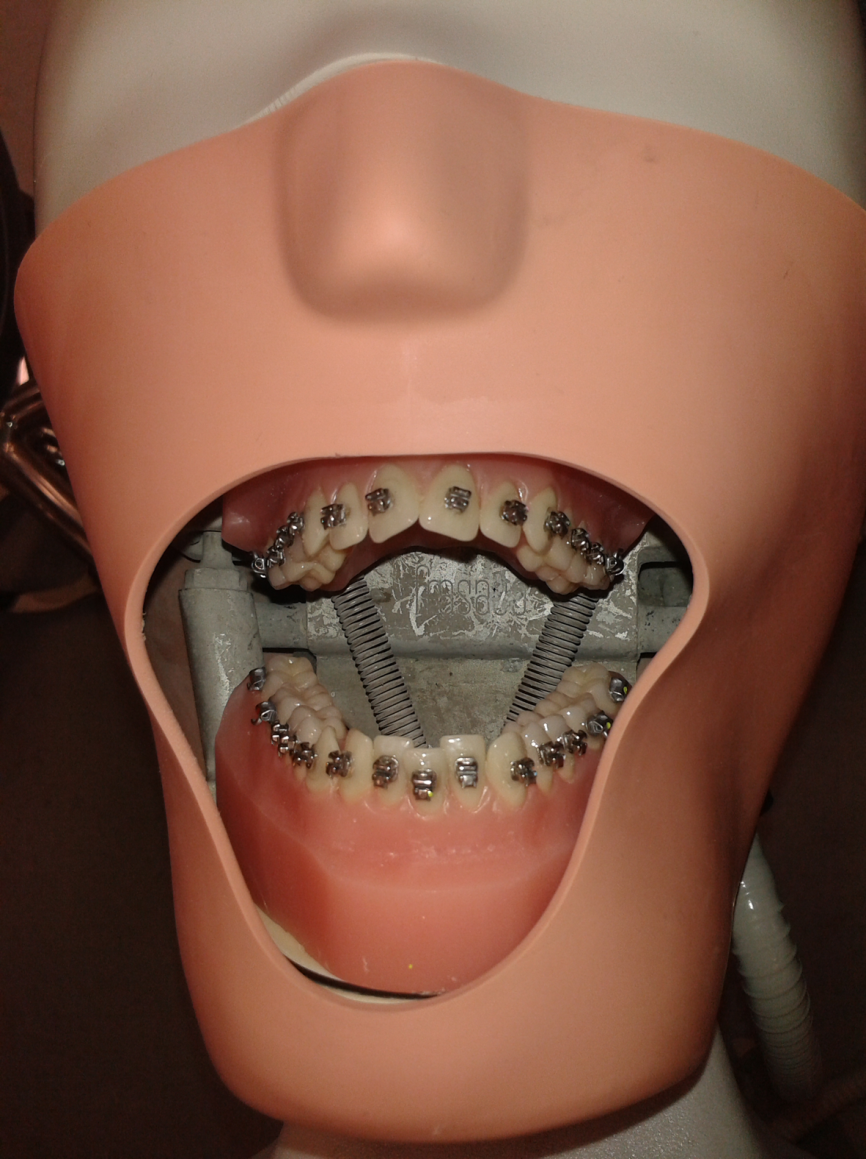


**Supporting figure 2**. Typodont positioned in a mannequin, to simulate the oral cavity environment.

| **Supporting table 1. Descriptive statistics of time spent (s) for both arches by all operators (n= 33) for the different bracket systems.** | | | | | |
| --- | --- | --- | --- | --- | --- |
| ***Treatment stage*** | ***Operation*** | ***Active SLB*** | ***Passive SLB*** | ***CB elastomeric ligatures*** | ***CB metallic ligatures*** |
| T0 | Archwire insertion | 64.6 ± 35.7 | 74.8 ± 32.6 | 64.1 ± 24.9 | 60.4 ± 33.4 |
|  | Ligation/Closure | 74.3 ± 22.8 | 55.4 ± 15.8 | 327.0 ± 139.9 | 539.1 ± 239.3 |
|  | Deligation/Opening | 54.9 ± 19.1 | 34.2 ± 6.9 | 87.5 ± 39.0 | 207.1 ± 88.9 |
|  | Archwire removal | 7.1 ± 4.2 | 6.6 ± 4.2 | 6.5 ± 4.8 | 10.2 ± 15.6 |
|  | **Total time** | 200.9 ± 64.5 | 171.1 ± 46.7 | 485.0 ± 185.9 | 816.8 ± 297.1 |
| T1 | Archwire insertion | 79.7 ± 39.5 | 67.1 ± 26.0 | 49.6 ± 19.3 | 54.3 ± 34.6 |
|  | Ligation/Closure | 64.4 ± 36.3 | 47.7 ± 21.7 | 288.2 ± 139.7 | 505.4 ± 224.0 |
|  | Deligation/Opening | 56.5 ± 38.7 | 35.4 ± 6.9 | 80.3 ± 34.7 | 195.5 ± 114.4 |
|  | Archwire removal | 18.5 ± 12.3 | 11.3 ± 10.0 | 10.1 ± 7.3 | 13.1 ± 10.5 |
|  | **Total time** | 219.1 ± 79.0 | 161.5 ± 42.2 | 428.3 ± 181.0 | 768.4 ± 328.6 |
| *Time in seconds; mean ± standard deviation. SLB, Self-ligating bracket; CB, conventional bracket.* | | | | | |

| **Supporting table 2. Descriptive statistics of time spent for the different bracket systems, for both arches, by different operator groups.** | | | | | | | |
| --- | --- | --- | --- | --- | --- | --- | --- |
| *Treatment stage* | *System* | *Operator group‡* | *Archwire insertion* | *Ligation/ Closure* | *Deligation/ Opening* | *Archwire removal* | *Total time* |
| T0 | Active SLB | Orthodontists | 57.7 ± 18.8 | 72.7 ± 21.4 | 49.6 ± 15.0 | 7.7 ± 4.9 | 187.8 ± 47.2 |
|  |  | Postgraduates | 62.4 ± 15.0 | 68.8 ± 14.5 | 59.5 ± 15.7 | 7.7 ± 5.1 | 198.4 ± 30.7 |
|  |  | Undergraduates | 73.7 ± 58.0 | 81.4 ± 30.1 | 55.4 ± 25.3 | 5.8 ± 1.8 | 216.4 ± 98.3 |
|  | Passive SLB | Orthodontists | 61.1 ± 20.5 | 50.3 ± 12.5 | 35.8 ± 9.7 | 5.6 ± 2.3 | 152.8 ± 29.3 |
|  |  | Postgraduates | 77.6 ± 34.8 | 55.0 ± 13.6 | 35.7 ± 4.9 | 8.4 ± 6.1 | 176.7 ± 49.1 |
|  |  | Undergraduates | 85.8 ± 37.6 | 60.8 ± 20.1 | 31.2 ± 4.4 | 5.8 ± 2.8 | 183.6 ± 55.8 |
|  | CB elastomeric ligatures | Orthodontists | 58.7 ± 25.0 | 240.9 ± 72.4 | 64.6 ± 9.5 | 5.7 ± 4.7 | 370.0 ± 97.5 |
|  |  | Postgraduates | 60.6 ± 19.8 | 276.4 ± 114.5 | 80.4 ± 24.5 | 9.1 ± 6.0 | 426.6 ± 151.1 |
|  |  | Undergraduates | 72.8 ± 29.0 | 463.6 ± 112.9 | 117.4 ± 50.6 | 4.6 ± 2.3 | 658.4 ± 163.8 |
|  | CB metallic ligatures | Orthodontists | 58.7 ± 38.4 | 448.9 ± 99.8 | 179.4 ± 43.2 | 6.7 ± 5.2 | 693.8 ± 138.5 |
|  |  | Postgraduates | 54.9 ± 28.5 | 421.4 ± 138.2 | 197.3 ± 52.4 | 19.1 ± 24.5 | 692.7 ± 173.6 |
|  |  | Undergraduates | 67.4 ± 34.6 | 747.1 ± 287.1 | 244.7 ± 134.7 | 4.7 ± 4.5 | 1064.0 ± 363.7 |
| T1 | Active SLB | Orthodontists | 78.2 ± 52.2 | 48.9 ± 15.0 | 50.0 ± 14.6 | 17.4 ± 11.6 | 194.4 ± 70.5 |
|  |  | Postgraduates | 91.3 ± 33.1 | 59.5 ± 21.7 | 55.8 ± 13.8 | 22.5 ± 15.8 | 229.2 ± 45.5 |
|  |  | Undergraduates | 69.5 ± 30.1 | 84.7 ± 52.7 | 63.7 ± 65.4 | 15.7 ± 8.5 | 233.7 ± 109.2 |
|  | Passive SLB | Orthodontists | 56.6 ± 17.4 | 40.5 ± 13.6 | 34.4 ± 7.0 | 11.6 ± 6.7 | 143.2 ± 33.6 |
|  |  | Postgraduates | 66.5 ± 22.0 | 41.7 ± 12.6 | 38.4 ± 8.2 | 12.7 ± 15.1 | 159.4 ± 36.2 |
|  |  | Undergraduates | 78.3 ± 33.4 | 60.8 ± 29.6 | 33.5 ± 4.5 | 9.4 ± 6.6 | 182.1 ± 49.1 |
|  | CB elastomeric ligatures | Orthodontists | 47.9 ± 16.6 | 211.4 ± 55.8 | 67.8 ± 14.0 | 11.3 ± 6.0 | 338.4 ± 75.0 |
|  |  | Postgraduates | 46.7 ± 20.5 | 223.5 ± 50.9 | 76.4 ± 17.6 | 12.2 ± 8.8 | 358.8 ± 81.4 |
|  |  | Undergraduates | 54.1 ± 21.3 | 429.6 ± 154.0 | 96.8 ± 53.5 | 7.0 ± 6.4 | 587.5 ± 224.8 |
|  | CB metallic ligatures | Orthodontists | 48.9 ± 25.3 | 392.5 ± 96.3 | 176.9 ± 58.4 | 14.2 ± 6.0 | 632.5 ± 156.4 |
|  |  | Postgraduates | 56.9 ± 47.2 | 402.2 ± 90.4 | 171.4 ± 57.4 | 11.1 ± 7.1 | 641.6 ± 175.1 |
|  |  | Undergraduates | 57.1 ± 30.3 | 721.6 ± 256.8 | 238.2 ± 179.4 | 14.0 ± 16.2 | 1030.9 ± 420.4 |
| *Time in seconds; mean ± standard deviation;* ‡*n= 11 per group. SLB, Self-ligating bracket; CB, conventional bracket.* | | | | | | | |
